# Identification and expression profile analysis of the SnRK2 gene family in cucumber

**DOI:** 10.7717/peerj.13994

**Published:** 2022-09-21

**Authors:** Zilong Wan, Shilei Luo, Zeyu Zhang, Zeci Liu, Yali Qiao, Xueqin Gao, Jihua Yu, Guobin Zhang

**Affiliations:** 1 Gansu Agricultural University, State Key Laboratory of Arid Land Crop Science, Lanzhou, China; 2 College of Horticulture, Gansu Agricultural University, Lanzhou, China

**Keywords:** Bioinformatics analysis, Cucumber, SnRK2 gene family, Gene expression

## Abstract

The sucrose non-fermenting-1-related protein kinase 2 (SnRK2) is a plant-specific type of serine/threonine protein kinase that plays an important role in the physiological regulation of stress. The objective of this study was to identify and analyze the members of the SnRK2 gene family in cucumber and lay a foundation for further exploration of the mechanism of *CsSnRK2* resistance to stress. Here, 12 *SnRK2* genes were isolated from cucumber and distributed on five chromosomes, phylogenetic clustering divided these into three well-supported clades. In addition, collinearity analysis showed that the *CsSnRK2* gene family underwent purifying selection pressure during evolution. *CsSnRK2* genes of the same group have similar exons and conserved motifs, and intron length may be a specific imprint for the evolutionary amplification of the *CsSnRK2* gene family. By predicting cis elements in the promoter, we found that the promoter region of *CsSnRK2* gene members had various cis-regulatory elements in response to hormones and stress. Relative expression analysis showed that *CsSnRK2.11* (group II) and *CsSnRK2.12* (group III) were strongly induced by ABA, NaCl and PEG stress; whereas *CsSnRK2.2* (group III) was not activated by any treatment. The response of group I *CsSnRK2* to ABA, NaCl and PEG was weak. Furthermore, protein interaction prediction showed that multiple CsSnRK2 proteins interacted with four proteins including protein phosphatase 2C (PP2C), and it is speculated that the *CsSnRK2* genes may also an independent role as a third messenger in the ABA signaling pathway. This study provides a reference for analyzing the potential function of *CsSnRK2* genes in the future research.

## Introduction

Biological and abiotic stressors greatly inhibit plant growth and development, severely affecting crop yield and quality, and, under various stress conditions, plants form complex response mechanisms. Among them, protein phosphorylation catalyzed by protein kinase is an important mechanism of signal transduction in plant cells (Huang et al., 2012; Yu, An & Li, 2014; Wang et al., 2001). The SnRK is a serine/threonine protein kinase that is widely present in plants. It can be divided into three subfamilies (SnRK1, SnRK2, and SnRK3) according to the conservation of the kinase activity domain (Halford, Boulyz & Thomas, 2000; Kulik et al., 2011; Umezawa et al., 2013). The abscisic acid (ABA) signaling pathway is a key pathway enabling plants to cope with adversity, and SnRK2 participates in the ABA signal transduction, forming the ABA-PYR-PP2C-SnRK2-downstream transcription factor coupling signaling pathway (Hrabak et al., 2003). In ABA deficiency, PP2C interacts with SnRK2 to inhibit the activity of related kinases, and the signaling pathway is closed. When ABA is present, its receptor binds to the relevant hormones and acts on PP2C, releasing the inhibition of phosphatase SnRK2 and opening the signaling pathway (Cheng et al., 2017; Sheard & Zheng, 2009). At present, owing to the important role of SnRK2 in different stress conditions, many plant-related SnRK2 gene families have been identified and studied; including *Arabidopsis thaliana* (10 *SnRK2s*) (Saha et al., 2014), rice (10 *SnRK2s*) (Saha et al., 2014), maize (11 *SnRK2s*) (Huai et al., 2008), soybean (22 *SnRK2s*) (Wei et al., 2017), cotton (20 *SnRK2s*) (Liu et al., 2017), wheat (10 *SnRK2s*) (Zhang et al., 2016), sorghum (10 *SnRK2s*) (Li-Bin et al., 2010), tomato (7 *SnRK2s*) (Sun et al., 2011), mungbean (8 *SnRK2s*) (Fatima et al., 2020), grapevine (8 *SnRK2s*) (Liu et al., 2016), pak-choi (13 *SnRK2s*) (Huang et al., 2015), and *Brassica napus* (114 *SnRK2s*) (Zhu et al., 2020).

The expression of the *SnRK2* gene derived from wheat is induced by ABA, drought, and other stresses (Holappa & Walker-Simmons, 1995; Liu et al., 2017). In *Arabidopsis*, except for *AtSnRK2.9*, the other nine *SnRK2* genes were found to be induced by mannitol and NaCl (Boudsocq, Barbier-Brygoo & Lauriere, 2004). *AtSnRK2.2-3* and *SnRK2.6* are core regulators of ABA signal transduction, and play key roles in coping with water stress and in controlling seed development and dormancy (Fujita, Yoshida & Yamaguchi-Shinozaki, 2013; Yasunari et al., 2009). All members of the SnRK2 gene family in rice can be induced by NaCl, among which *SAPK8*, *SAPK9*, and *SAPK10* are induced by ABA (Kobayashi et al., 2004). Among the 11 *ZmSnRK2* genes in maize, *ZmSnRK2.3/6 w* as strongly induced by NaCl treatment, while most of the other genes were weakly induced by salt stress; *ZmSnRK2.3/7* was strongly induced by low-temperature treatment (Huai et al., 2008). Plants overexpressing *ZmSnRK2.11* showed reduced sensitivity to salt and drought under salt and drought stress treatments (Zhang et al., 2015). Compared with normal growing plants, the overexpression of the *TaSNRK2.4* gene prolonged the growth cycle and increased the yield of wheat plants. Additionally, *Arabidopsis* plants have an increased resistance to drought, high salinity and low temperatures (Mao et al., 2010). Recent studies have shown that *SnRK2* regulates a variety of plant growth and development processes in the ABA signaling pathway, including stomatal opening and closing, seed dormancy and germination, and the flowering cycle (Fujii & Zhu, 2009; Wang et al., 2013). For example, the *SnRK2* gene in grapes is involved in the stomatal regulation of osmotic stress (Boneh et al., 2012). In summary, different *SnRK2* genes are involved in the response of plants to various stressors.

Cucumber is an important vegetable cash crop, that has significant production and economic value worldwide (Wen et al., 2016). Currently, drought and other adverse stresses conditions have serious effects on the growth, development, photosynthesis, yield, and quality of cucumber. Transcriptome and transgenic analyses have shown that many cucumber genes are abnormally expressed under salt and drought stress (Du et al., 2021; Ouzounidou et al., 2016; Wang et al., 2012). Therefore, improving the cucumber yield and quality by increasing their resistance to stress is a particularly important initiative. In this study, 12 cucumber SnRK2 gene family members were identified, as well as systematically and bioinformatically analyzed. In addition, we analyzed the relative gene expression of cucumber under normal environmental conditions and several stressors. This study provides a theoretical basis of the functions of this gene family for further studies.

## Material and Methods

### Plant materials

Cucumber seeds L306 (Cucumis sativus L, “L306” cultivar) were used in this experiment. The seeds were disinfected with 5% sodium hypochlorite and then germinated until the two true leaves were completely expanded. The seedlings were cultured in Yamazaki cucumber nutrient solution at 25 °C/18 °C (day/night), 250 µmol.m^−2^ s^−1^ light and 70% relative humidity in an artificial climate chamber. Samples were collected at three true leaf stages. This experiment set up four treatments: 50 µM ABA, 100 µM ABA, 200 mM NaCl and 10% PEG. In each treatment, a total of 20 seedling samples of similar size were treated. Five sampling time points were set for different treatments, and the leaves of four seedlings were collected at each time point (0, 3, 6, 12, and 24 h). RNA was extracted after cutting and mixing, and three CT values were measured as repetition.

### Identification of CsSnRK2 genes

To screen candidate SnRK2 genes in cucumber, BLASTp was used to compare the protein sequences of 10 *SnRK2s* of *Arabidopsis* with that of cucumbers using an online database (http://cucurbitgenomics.org/). Set the BLASTp e-value to 1e^−10^ (Altschul, 1997). The redundancy of the results was removed manually. The hidden Markov model (HMM) of the kinase domain (PF00069.25) was obtained from the Pfam database (http://pfam.xfam.org/), and the cucumber genome database was further searched by HMMER 3.0 software. TBtools was also used to screen candidate genes; Pfam (http://pfam.xfam.org/search#tabview=tab1) and NCBI-CDD (https://www.ncbi.nlm.nih.gov/cdd/) databases were used for validation and candidate genes without SnRK2 domains (registration number: PF00069.25) were manually deleted (Zhou et al., 2018).

### Chromosome localization and basic information analysis

For the distribution on chromosomes, the gff3 file of ”9930” V3 version of China long was downloaded. SnRK2 genome location information was obtained by screening. It was named *CsSnRK2* s according to its distribution on chromosomes. The chromosome map of the gene was drawn by MG2C (http://mg2c.iask.in/mg2c_v2.0/). WoLF PSORT (https://wolfpsort.hgc.jp/) was used to predict protein subcellular localization (Xiong et al., 2015). The number of amino acids as well as the molecular weights, isoelectric points, and other physical and chemical information of *CsSnRK2* gene were analyzed on ExPaSy website (https://web.expasy.org/protparam/) (Liu et al., 2017). The secondary structure of the cucumber SnRK2 protein was predicted by using the PRABI website (https://npsa-prabi.ibcp.fr/cgi-bin/npsa_automat.pl?page=npsa_sopma. html).

### Collinearity analysis of *CsSnRK2* genes

Collinearity analysis using MCScanx provided by TBtools software. Cucumber genome was compared with *Arabidopsis* genome, maize genome and rice genome by BLASTp, and the search threshold was set as e-value <1e^−5^. Other default parameters have not been modified. The results of genome-wide BLASTp were used to calculate all possible collinear pairs between chromosomes. Using TBtools to map the collinear pairs of *SnRK2* genes in cucumber, *Arabidopsis*, maize and rice (Chen et al., 2020). The protein sequences of the gene pairs were extracted for multiple sequence alignment, and then the alignment results and the CDS sequences of the gene pairs were used to calculate the non-synonymous/synonymous (d_N_/d_S_) values of duplicate gene pairs using PAL2NAL (http://www.bork.embl.de/pal2nal/index.cgi?) (Wang et al., 2021).

### Phylogenetic, structure and conserved motif analysis

The phylogenetic trees of SnRK2 gene family of cucumber, *A.thaliana*, rice, maize, sugar beet, tomato, grapevine and pak-choi were constructed by using MEGA 11 software with ClustalW methods to align multiple sequence (Kumar, Stecher & Tamura, 2016). The bootstrap values of 1000 replicates were calculated at each node. The minimum neighbor method was selected to represent the phylogenetic tree. Then, the phylogenetic tree was visualized using the iTOL online tool (http://itol.embl.de/) (Ivica & Peer, 2016). The CDs and whole genome sequences of 12 cucumber *SnRK2* genes were uploaded to GSDS (http://gsds.cbi.pku.edu.cn/) to visualize the positions of introns and exons and untranslated regions (Hu et al., 2014). We analyzed the conserved motifs of gene by MEME (http://meme-suite.org/tools/meme). The maximum motif number was set to 10, and the other parameters are the default values (Bailey et al., 2009).

### Analysis of cis-acting elements, tissue-specific expression and protein interaction networks

TBtools were used to extract the genomic sequence 2,000 bp upstream of the start codon (ATG) of *SnRK2* genes in cucumber. Then, the 2000 bp sequences of 12 *CsSnRK2* were submitted to PlantCARE (http://bioinformatics.psb.ugent.be/webtools/plantcare/html/) for prediction. The RNA-Seq data of cucumber in different tissues and organs (tendril-base, tendril, root, leaf, stems, ovary-unfertilized, ovary-fertilized, ovary) were obtained from the cucumber genome data using the registration number PRJNA80169 (http://cucurbitgenomics.org/). The data were converted by log2 method, and TBtools software was used to draw the heat map of *CsSnRK2* genes expression. Using 12 CsSnRK2 protein sequences as targets, the protein-protein interaction network was predicted by using STRING website (https://string-db.org/cgi).

### RNA extraction and Real-Time PCR (qRT-PCR)

Total RNA was isolated from collected samples using a Plant RNA Extraction kit (Tiangen, China). cDNA was synthesized with fastking cDNA dispersion RT supermaxs Kit (Tiangen, China) and 2uL RNA was used as template. Design of primers using CDs sequences of genes. The SYBR Green kit (Tiangen, China) was used for fluorescence quantitative analysis. The volume of reaction system was 20 uL, which contained 2 uL cDNA solution, 10 uL 2*SuperReal PreMix Plus, 0.6 uL of 10 uM forward and reverse primers, 0.4 uL 50*ROX Reference Dye and 6.4 uL of distilled deionized water. qRT-PCR analysis using LightCycler^®^ 480 II real-time fluorescent quantitative PCR instrument. Each qRT-PCR reaction was carried out with three technical replicates. Cucumber actin (DQ115883) was used as internal control. The amplification primers and internal reference primers are shown in Table 1. Amplification conditions were as follows: 95 °C for 15 min, and 40 cycles of 95 °C for 10 s and 60 °C for 30 s. The relative expressions levels of the *SnRK2* genes were calculated using the 2^−ΔΔCt^ method (Livak & Schmittgen, 2002). SPSS 20.0 software was used for one-way ANOVA, and Duncan method was used for significance test (*p* < 0.05). Using origin 9.0 software to complete the histogram of relevant expression, and the values of the last drawn graph are the average values of three repetitions.

**Table 1:** Primer sequences for qRT-PCR.

**Gene Name**	**Forward Primer Sequence (5′–3′)**	**Reverse Primer Sequence (5′–3′)**
*CsSnRK2.1*	GCAGATCATCGAGGAAGCGTCAG	CAAGTCCAGTTCAGCATCCGTCTC
*CsSnRK2.2*	TTATGCACGACAGTGACCGCTATG	GTCTCTCATCAACCTGGCAACCC
*CsSnRK2.3*	TTGGTTGGAGCATACCCGTTTGAG	GCGAGAAAGAAGGTTGCGACATTC
*CsSnRK2.4*	TCATCCTTGCTGCATTCGAGACC	CCACACGACCATACATCTGCCATC
*CsSnRK2.5*	TTAGAAGAAAGCAAAGTCCC	ATGTGGTCGGAAGTGAGAAG
*CsSnRK2.6*	CGTTGGTACTCCGGCATACATAGC	CCACACGACCATACATCTGCCATC
*CsSnRK2.7*	TAGTTGGAGGGCAAGGGCAATTTG	CCAAGGCACAAACAAAGTCACCAC
*CsSnRK2.8*	GGATCGCTCCGCACTTACTGTTG	ACCGAAGTTTCCAGAGCCAATGTC
*CsSnRK2.9*	AGATTGTGAGGGAGGCAAGAAAGC	GTCGTGATCCTCTTCGGTGTTCG
*CsSnRK2.10*	AACTCAGCCGCCTCGCAGTC	CCTCCGGTGCTGTGTATGTTGC
*CsSnRK2.11*	CAGACCGAAGAAGTTGTTGCCATG	ACCAAAGCCGCGTGGGTTTTG
*CsSnRK2.12*	GCCCAACCATCAAAACCCATCATG	TCCTCTTGTTCCAGCCCAGTCTC
*actin*	GCCCTCCCTCATGCCATTCT	TCGGCAGTGGTGGTGAACAT

## Results

### Chromosome distribution and basic information of *CsSnRK2* genes

To explore the function of the SnRK2 gene, we systematically analyzed the physicochemical properties of the cucumber SnRK2 protein. We identified 12 *CsSnRK2* genes and named them sequentially from *CsSnRK2.1* to *CsSnRK2.12*. The *SnRK2* genes differed substantially according to the encoded protein size and biophysical properties (Table 2). The number of amino acids ranged from 298 to 673. The sequence of *CsSnRK2.6* protein was the longest, containing 673 amino acids, while the sequence of *CsSnRK* 2.9 protein was the shortest, containing 298 amino acids. The molecular weight of *CsSnRK2* ranged from 34,754.37 to 77,009.99 Da, and the theoretical isoelectric point (pI) ranged from 4.71 to 8.8. *CsSnRK* 2.5 and *CsSnRK* 2.7 were basic proteins, whereas the others were acidic proteins. The total average hydrophobic index of the 12 CsSnRK2 gene family members was negative, indicating that they were hydrophilic proteins.

**Table 2:** *CsSnRK2* genes family members and characteristics.

**Gene ID**	**Gene name**	**Chromosom**	**Gene location**		**Amino acid number**	**Molecular weight/Da**	**pI**	**Instability index**	**Aliphatic index**	**Grand average of hydropathicity**
			**Start position**	**End position**						
CsaV3_2G014200.1	*CsSnRK2.1*	2	11843438	11848872	361	41238.84	4.71	53.36	89.89	−0.301
CsaV3_2G016930.1	*CsSnRK2.2*	2	14181637	14188320	363	41074.59	4.68	36.96	87.02	−0.286
CsaV3_3G002220.1	*CsSnRK2.3*	3	1788904	1793145	340	38382.87	5.76	35.92	90	−0.306
CsaV3_4G030050.1	*CsSnRK2.4*	4	19803919	19811002	355	40895.4	5.99	45.06	79.61	−0.565
CsaV3_4G030060.1	*CsSnRK2.5*	4	19819061	19836491	543	62061.34	7.79	47.81	84.2	−0.456
CsaV3_4G030080.1	*CsSnRK2.6*	4	19839540	19846552	673	77009.99	6.56	46.78	85.88	−0.423
CsaV3_4G030100.1	*CsSnRK2.7*	4	19856029	19860650	372	42436.16	8.8	40.48	77.82	−0.558
CsaV3_4G030120.1	*CsSnRK2.8*	4	19871598	19886067	486	55726.63	5.53	46.09	83.6	−0.409
CsaV3_4G030140.1	*CsSnRK2.9*	4	19893248	19896492	298	34754.37	6.09	52.65	71.98	−0.712
CsaV3_4G030260.1	*CsSnRK2.10*	4	19973756	19978649	344	38949.36	5.24	36.12	90.67	−0.293
CsaV3_6G045260.1	*CsSnRK2.11*	6	26819227	26822605	355	40935.31	5.67	45.71	79.61	−0.579
CsaV3_7G008840.1	*CsSnRK2.12*	7	5514315	5517582	365	41222.74	4.77	37.92	90.55	−0.314

Subcellular localization prediction (Table 3) results showed that all members of the cucumber SnRK2 gene family were localized in the cytoplasm, and most were also localized in the nucleus. The predicted secondary structure of the cucumber SnRK2 protein was mainly composed of an *α*-helix (36.05%–44.97%) and irregular curl (36.10%–45.43%), and the extended structure (9.40%–20.99%) and ß-turn (3.76%−7.37%) were relatively small.

**Table 3:** Secondary structure and subcellular localization of *CsSnRK2* genes.

**Gene ID**	**Gene name**	***α*-helix %**	**Beta turn %**	**Random coil %**	**Extended strand %**	**Subcellular localization**
CsaV3_2G014200.1	*CsSnRK2.1*	38.50	5.54	38.50	17.45	Cytoplasmic, Cytoskeleton
CsaV3_2G016930.1	*CsSnRK2.2*	36.09	6.61	41.32	15.98	Cytoplasmic, Chloroplast
CsaV3_3G002220.1	*CsSnRK2.3*	39.41	4.41	40.29	15.88	Cytoplasmic, Nuclear
CsaV3_4G030050.1	*CsSnRK2.4*	41.41	5.35	38.03	15.21	Cytoplasmic, Cytoskeleton
CsaV3_4G030060.1	*CsSnRK2.5*	35.54	7.37	36.10	20.99	Cytoplasmic, Nuclear
CsaV3_4G030080.1	*CsSnRK2.6*	41.60	4.90	36.85	16.64	Cytoplasmic, Nuclear
CsaV3_4G030100.1	*CsSnRK2.7*	34.41	3.76	45.43	16.40	Cytoplasmic, Nuclear
CsaV3_4G030120.1	*CsSnRK2.8*	41.15	3.70	39.71	15.43	Cytoplasmic, Nuclear
CsaV3_4G030140.1	*CsSnRK2.9*	44.97	3.69	41.95	9.40	Cytoplasmic, Nuclear
CsaV3_4G030260.1	*CsSnRK2.10*	43.38	6.20	35.21	15.21	Cytoplasmic, Cytoskeleton
CsaV3_6G045260.1	*CsSnRK2.11*	36.05	5.52	41.57	16.86	Cytoplasmic, Nuclear
CsaV3_7G008840.1	*CsSnRK2.12*	36.44	6.03	40.55	16.99	Cytoplasmic, Chloroplast

**Figure 1:**
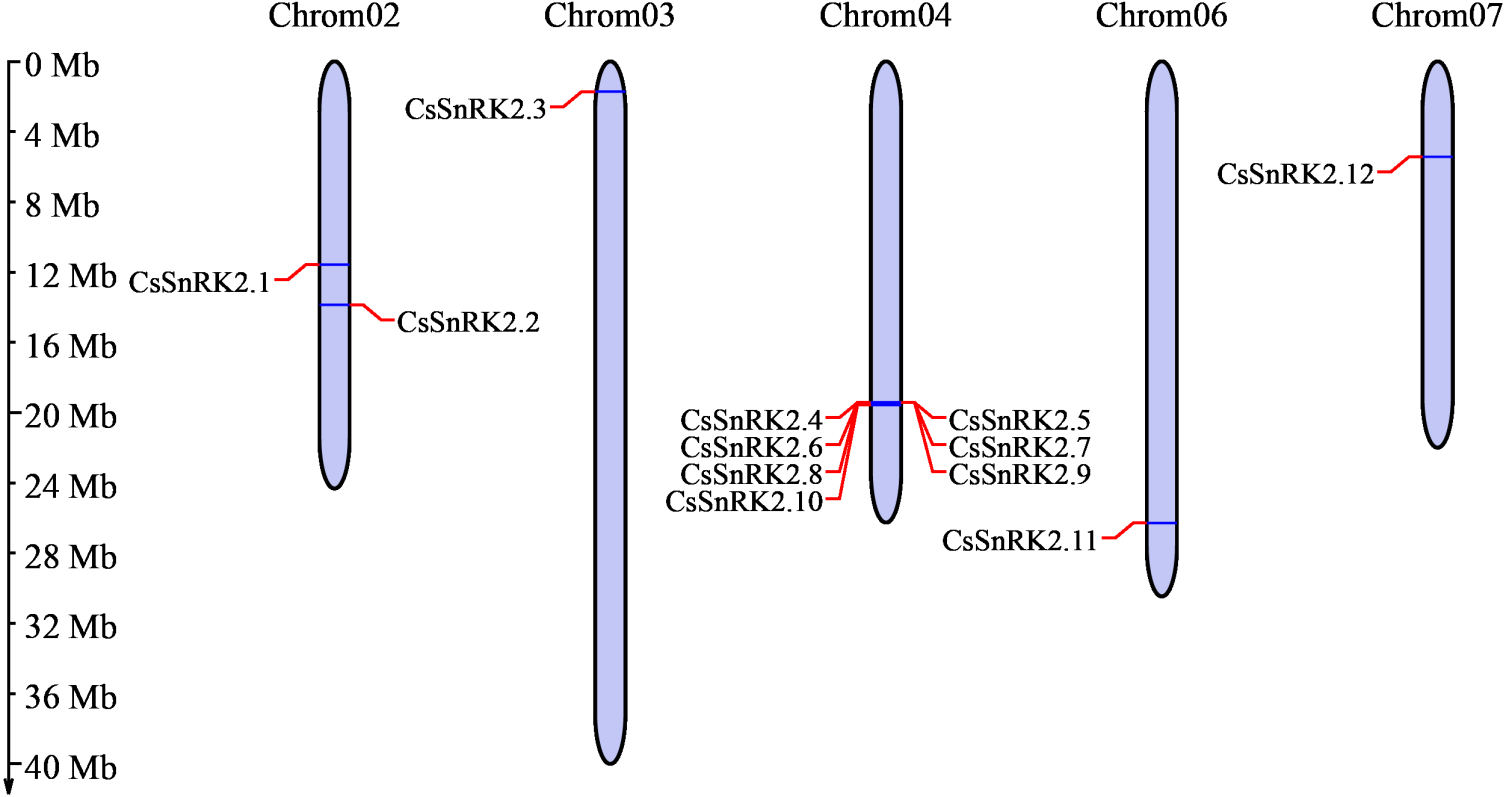
Distribution of *CsSnRK2* genes family on chromosome. Chrom01-07 are chromosome names. The chromosome scale on the left is in millions of bases (Mb).

Chromosome analysis of the cucumber SnRK2 gene family showed that 12 *CsSnRK2* genes were unevenly distributed on five cucumber chromosomes (Fig. 1). There were two genes distributed on chromosome 2 (*CsSnRK2.1* and *CsSnRK2.2*) and one gene each on chromosomes 3 (*CsSnRK2.3*), 6 (*CsSnRK2.11*), and 7 (*CsSnRK2.12*). The other genes were located on chromosome 4 (*CsSnRK2.4*-*CsSnRK2.10*). No members of this gene family were found on chromosomes 1 or 5.

### Collinearity and selective pressure analysis of the *CsSnRK2* genes

**Figure 2:**
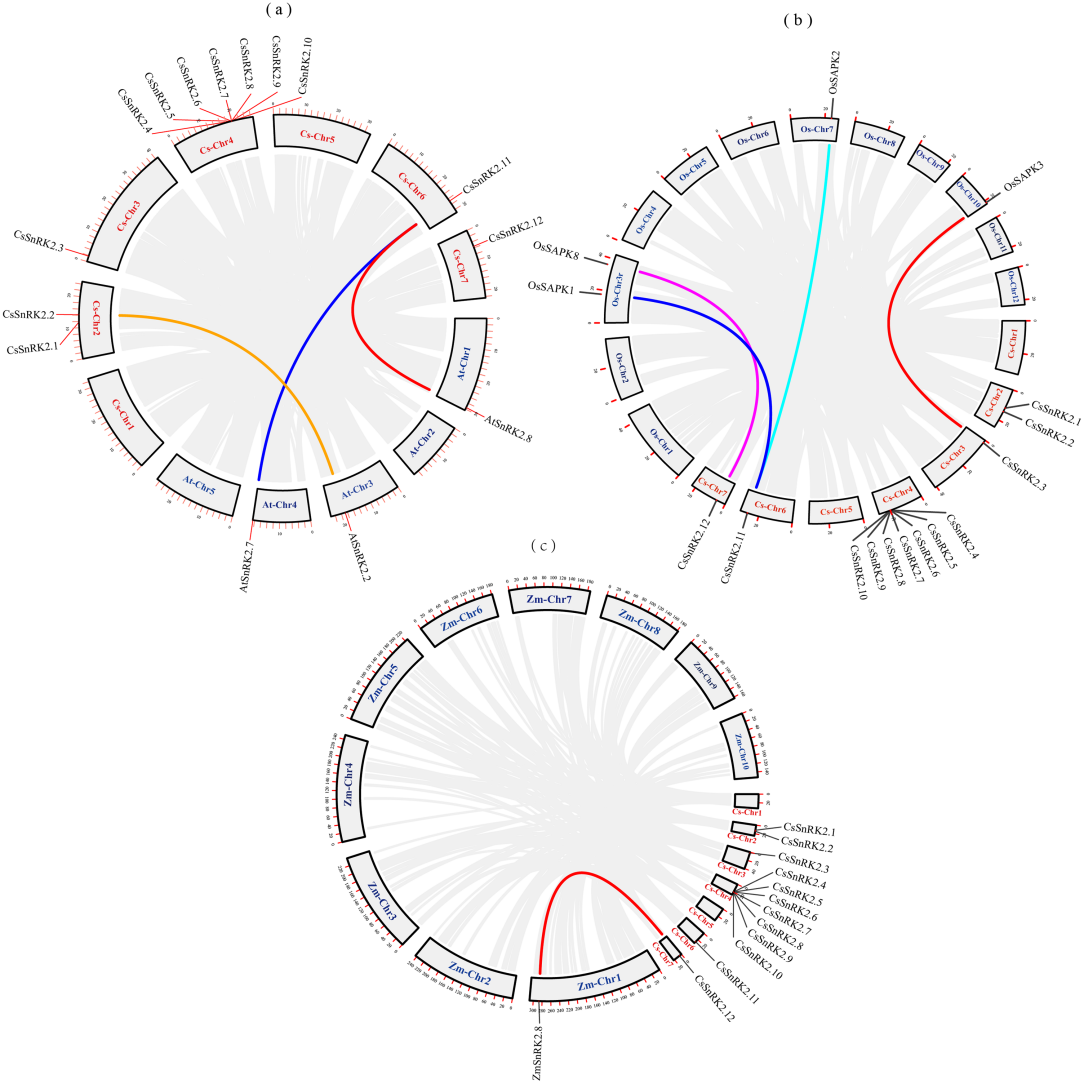
Collinearity analysis of cucumber with *Arabidopsis*, rice and maize. The gray rectangles represent chromosomes. The chromosome of cucumber (Cs-Chr1-7) is shown in red. *Arabidopsis* chromosomes (At-Chr1-5), rice chromosomes (Os-Chr1-12) and maize chromosomes (Zm-Chr1-10) are shown in blue. The gray lines represent collinear blocks of the *SnRK2* gene, and the colored lines represent collinear pairs of the *SnRK2* gene.

**Figure 3:**
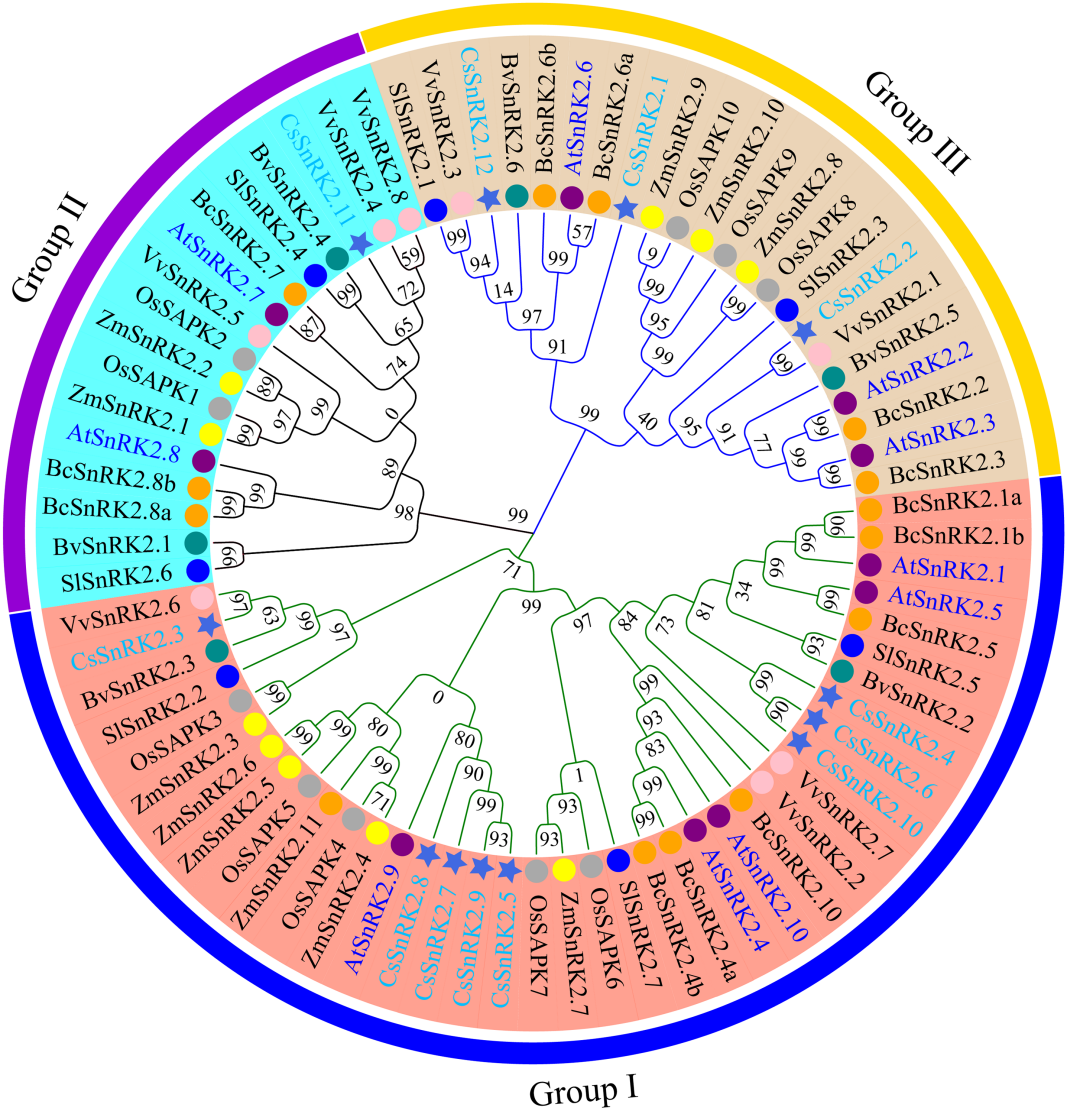
Phylogenetic analysis of the SnRK2s family genes from cucumber, *A.thaliana*, rice, maize, sugar beet, tomato, grapevine, and pak-choi. Royal blue star represent cucumber, purple circles represent *A.thaliana*, dark gray circles represent rice, yellow circles represent maize, dark cyan circles represent sugar beet, blue circles represent tomato, pink circles represent grapevine, orange circles represent pak-choi. phylogenetic tree was constructed using the minimum neighbor method by MEGA 11. The bootstrap values of 1000 replicates were calculated at each node. Lines and arcs with different colors represent different subfamilies.

**Figure 4:**
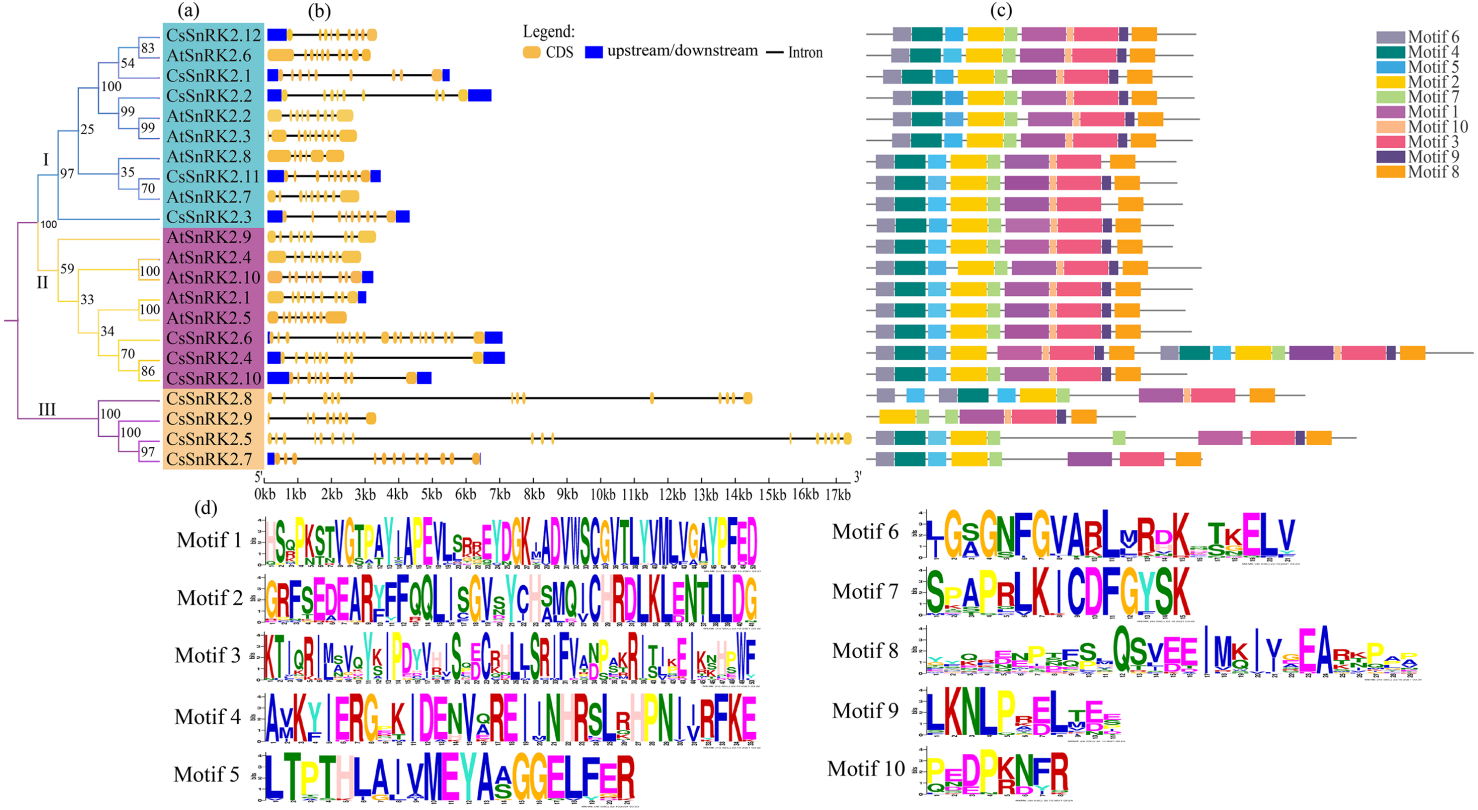
Phylogenetic tree, structure and conserved motif relationship of *CsSnRK2* and *AtSnRK2* genes. (A) The SnRK2 phylogenetic tree is divided into three groups (I. II, and III), and different colors represent different branches. (B) The blue boxes, black lines, and yellow ellipse in the gene structural diagram represent untranslated region, introns, and CDS sequence, respectively. (C) Analysis with MEME to investigate 10 conserved motifs of SnRK2 proteins. Motifs are indicated by 10 different colored boxes. (D) Basic composition of 10 conserved motifs.

We explored the evolutionary process of *CsSnRK2*, *AtSnRK2*, *ZmSnRK2*, and *OsSAPK2* genes. Collinearity analysis using MCScanx was performed using the TBtools software (Fig. 2). There were three pairs of collinearity genes between cucumber and *A.thaliana*, four pairs between cucumber and rice, and one pair between cucumber and maize. The col-linearity of the *AtSnRK2, OsSAPK, ZmSnRK2,* and *CsSnRK2* genes could be broadly classified into two categories; one-to-many (*CsSnRK2.11*-*AtSnRK2.7/8, CsSnRK2.11*-*OsSAPK1/2*), or one-to-one (*CsSnRK2.2*-*AtSnRK2.2, CsSnRK2.12*-*OsSAPK8, CsSnRK2.3*-*OsSAPK3, CsSnRK2.12*- *ZmSnRK2.8*). In addition, these gene pairs belonged to the same group in the phylogenetic tree (Fig. 3), and their gene structure and conserved motifs were highly similar (Fig. 4).

Purifying selection is the process of removing unfavorable mutations, whereas positive selection is the accumulation of new favorable mutations and their transmission to the entire population (Qiao et al., 2015). The value of d_N_/d_S_ plays a crucial role in gene selection and evolution; d_N_/d_S_ >1 is positive selection, d_N_/d_S_ = 1 is neutral selection, 0 < d_N_/d_S_ <1 is purifying selection (Yadav et al., 2015). The results showed that all three pairs of genes had d_N_/d_S_ values <1; indicating that the evolutionary process underwent purifying selection pressure and evolved conservatively, which helped maintain the basic functions of the genes (Table 4).

**Table 4:** Selective pressure analysis of *CsSnRK2* genes family.

**A pair of genes**	**S**	**N**	**d**_**S**_	**d**_**N**_	**d**_**N**_ **/d**_**S**_
*AtSnRK2.2∼CsSnRK2.2*	254.2	768.8	3.5115	0.1588	0.0452
*AtSnRK2.7∼CsSnRK2.11*	235.8	766.2	4.4248	0.1547	0.0350
*AtSnRK2.8∼CsSnRK2.11*	241.1	841.9	2.2890	0.0930	0.0406
*OsSAPK1∼CsSnRK2.11*	247.7	775.3	5.7825	0.1281	0.0222
*OsSAPK2∼CsSnRK2.11*	199.0	590.0	6.7995	0.1378	0.0203
*OsSAPK3∼CsSnRK2.3*	250.4	751.6	4.7973	0.1483	0.0309
*OsSAPK8∼CsSnRK2.12*	242.7	807.3	6.9083	0.0831	0.0120
*ZmSnRK2.8∼CsSnRK2.12*	265.8	817.2	11.7553	0.0894	0.0076

**Notes.**

* “S” represents the numbers of synonymous, “N” represents non-synonymous, “d_S_” represents the synonymous mutation fre-quency and “d_N_” represents the non-synonymous mutation frequency and the ratio of d_N_/d_S_.

### Phylogenetic tree analysis of the *CsSnRK2* genes

To explore the functional characteristics and evolutionary relationships of the *CsSnRK2* genes, a phylogenetic tree was constructed using multiple sequences alignments of SnRK2 protein sequences from the 12 cucumber, 10 *Arabidopsis*, 10 rice, 11 maize, six sugar beet, seven tomato, eight grapevine, and 13 pak-choi genes. The phylogenetic tree showed that the SnRK2 gene family was clustered into three branches, labeled as group I, group II, and group III (Fig. 3), which is consistent with the results of previous studies in *Arabidopsis* (Umezawa et al., 2009). The cucumber SnRK2 family members of cucumbers were unevenly distributed among the three groups. Group I had eight members, group II had one member, and group III had three members. The phylogenetic tree showed that *CsSnRK2* was more closely grouped with *SnRK2* from the dicots *Arabidopsis,* sugar beet, tomato, grapevine, and pak-choi than with *SnRK2* from the monocots rice and maize. The cucumber SnRK2 and grapevine SnRK2 proteins were highly homologous, indicating that they have a close phylogenetic relationship and conservative evolution. Specifically, *CsSnRK2.3* and *VvSnRK2.6*; *CsSnRK2.11* and *VvSnRK2.4/8*; and *CsSnRK2.2* and *VvSnRK2.1* had the closest phylogenetic relationships, indicating that they may have the most similar functional characteristics.

### Structure and conserved motif analysis of the *CsSnRK2* genes

Analysis of the gene structure and conserved motifs of cucumber *SnRK2* provided important information for understanding the evolution of this gene (Fig. 4). Therefore, a phylogenetic tree was constructed using the *CsSnRK2* and *AtSnRK2* genes. According to the phylogenetic tree, the cucumber and *Arabidopsis* SnRK2 gene families could be divided into three groups (Fig. 4A). In the third group, all cucumber genes differed from the previous (Fig. 3). The exon-intron distribution of cucumber *SnRK2* genes was conserved, most of these had nine exons and eight introns; this was consistent with most *Arabidopsis SnRK2* genes, except for *AtSnRK2.6* (10 exons), *AtSnRK2.3* (10 exons), and *AtSnRK2.8* (6 exons) (Fig. 4B) (Yoon et al., 1997). In addition, the number and location of introns and exons in the third group of *CsSnRK2* were different from those in *Arabidopsis* homologs, which may contribute to the functional diversification of *CsSnRK2*. Moreover, most introns of the cucumber *SnRK2* gene were found to be much longer than those in the *Arabidopsis SnRK2* genes.

We used TBtools to predict the conserved motifs in the *CsSnRK2* and *AtSnRK2* families (Fig. 3C). A total of 10 conserved motifs were identified, and their motif sequences are shown in Fig. 4D. Except for *AtSnRK2.3*, all genes had motifs 1-10, and the sequence and position of the motif in the structure were nearly the same, showing high conservatism in evolution. In the third group, there were significant differences in motif placement and location, and motif deletions were also observed. For example, *CsSnRK2.7* and *CsSnRK2.8* lacked motif 9, while *CsSnRK2.9* lacked motif 4/5/6 but hads two of motif 1, which was similar to that of *CsSnRK2.5*. We hypothesized that these differences were associated with gene-specific functions.

### Cis-acting element of the *CsSnRK2* promoter region

**Figure 5:**
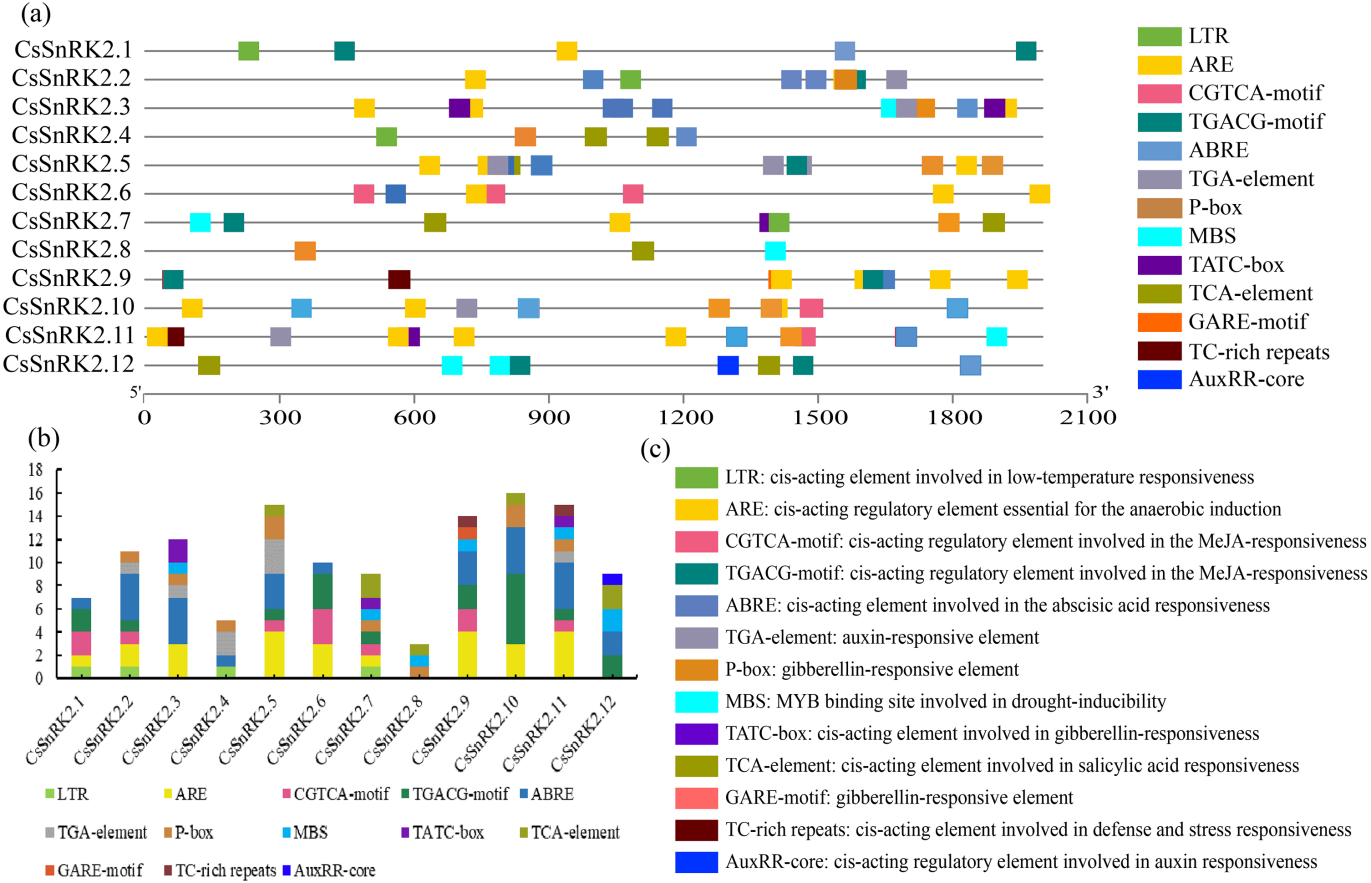
The 2 kb upstream region cis-acting element of SnRK2 gene family in cucumber. (A) The distribution of cis-acting elements in the promoter sequence of *CsSnRK2* genes are represented by different colors. (B) Number of cis-acting elements statistics. (C) Cis-element names and their functional notes.

The plant SnRK2 gene family plays an important role in abiotic stress responses, and the analysis of regulatory elements provides favorable information for the study of gene function. Several cis elements were identified in the promoter regions of the *CsSnRK2* genes (Fig. 5). Among them, at large variety of hormone-related cis elements, such as P-box, TATC-box, GARE-motif, TCA-element, ABRE, TGACG-motif, CGTCA-motif, TGA-element, and AuxRR-core, indicated that they responded to different hormones such as MeJA, growth hormone, ABA, ethylene, GA and SA. The four cis elements were MBS, LTR, TC-rich repeats and ARE, which are involved in drought, low temperature, defense, stress and anaerobic induction. In addition, six cis elements associated with development were identified in the *CsSnRK2* promoter, including P-box, TATC-box, Gare-Motif, ABRE, and TGA-element/AuxRR-core. The *CsSnRK2.8* gene had the least active elements, containing only three elements, while the other genes had at least five elements. The genes, except *CsSnRK2.7* and *CsSnRK2.8*, contained ABRE elements. This suggests that most *CsSnRK2* genes are responsive to ABA. Only four members (*CsSnRK2.1, CsSnRK2.2, CsSnRK2.4,* and *CsSnRK2.7*) contained the LTR-element. In addition, two members (*CsSnRK2.9,* and *CsSnRK2.11*) contained TC-rich repeats, and five members (*CsSnRK2.3, CsSnRK2.7, CsSnRK2.8, CsSnRK2.11,* and *CsSnRK2.12*) contained MBS. We hypothesized that exogenous stress could induce *CsSnRK2* gene expression through cis-responsive elements, thereby enhancing plant resistance to adversity.

### Tissue-specific expression analysis of the *CsSnRK2* genes

**Figure 6:**
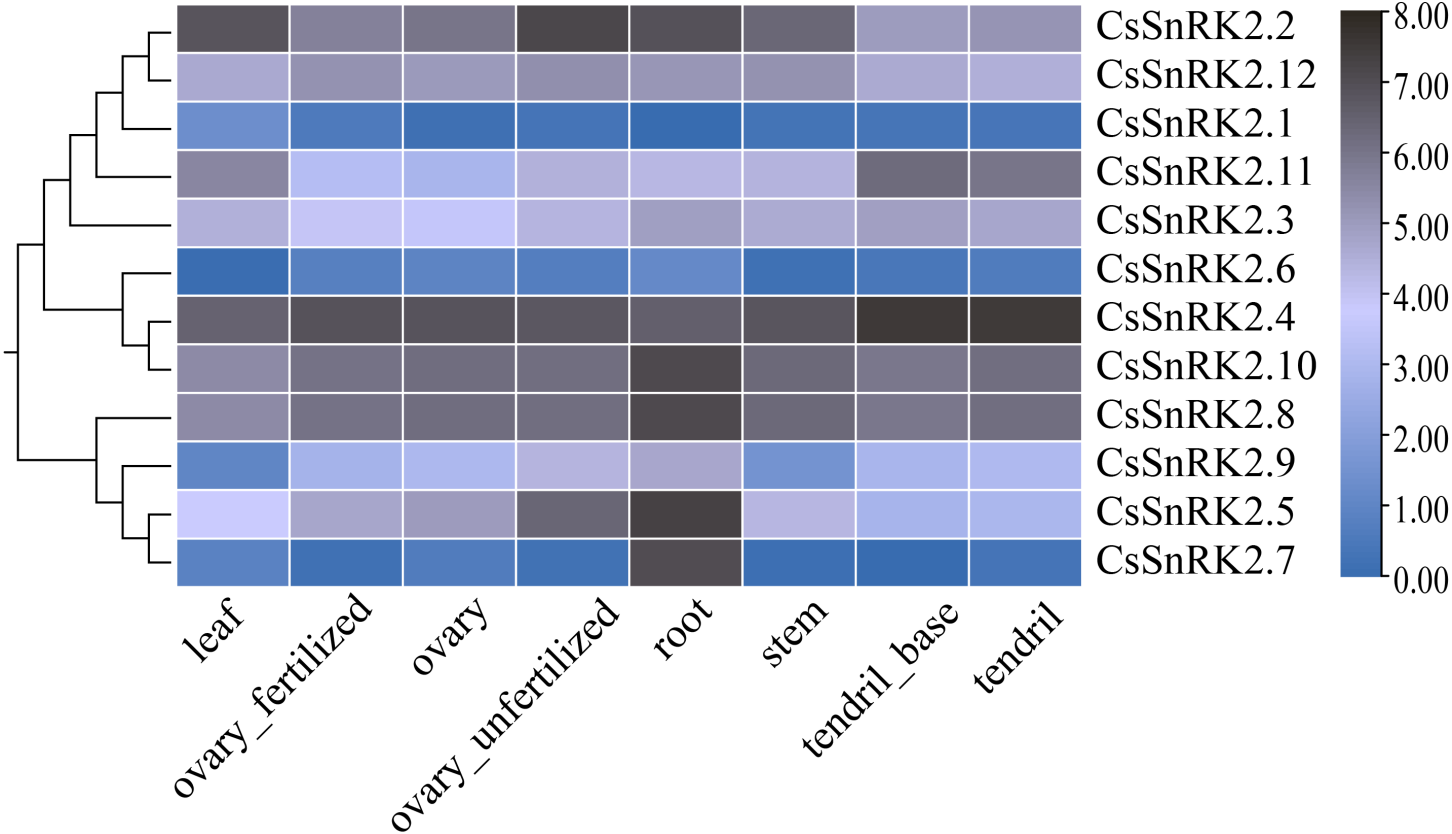
Heat map of tissue-specific RNA-Seq data expression of *SnRK2* genes in Cucumber. The *CsSnRK2* genes were listed on the right side of the expression array, and the color bar to the right of the genes are a scaled log2 expression value based on RNA-Seq data, indicating a color gradient from low expression (light blue) to high expression (black).

The heat map of RNA-Seq data expression of the *CsSnRK2* genes in different tissues and organs using TBtools software (Fig. 6) revealed that *CsSnRK2.1* and *CsSnRK2.6* were not expressed in almost all tissues. *CsSnRK2.2*, *CsSnRK2.4*, *CsSnRK2.8*, *CsSnRK2.10* and *CsSnRK2.12* were strongly expressed in each tissue, and their function may be related to growth and development. *CsSnRK2.5* was strongly expressed in the roots and moderately expressed in unfertilized ovaries. *CsSnRK2.7* was strongly expressed in the roots and weakly expressed in other tissues. It was speculated that *CsSnRK2.5* and *CsSnRK2.7* may play an important role in root growth. *CsSnRK2.11* was highly expressed in tendrils and the base of tendrils, and moderately expressed in other tissues. *CsSnRK2.3* was moderately expressed in all the tissues. *CsSnRK2.9* was moderately expressed in the roots and weakly expressed in the leaves.

### qRT-PCR expression characteristics of the *CsSnRK2* genes

**Figure 7:**
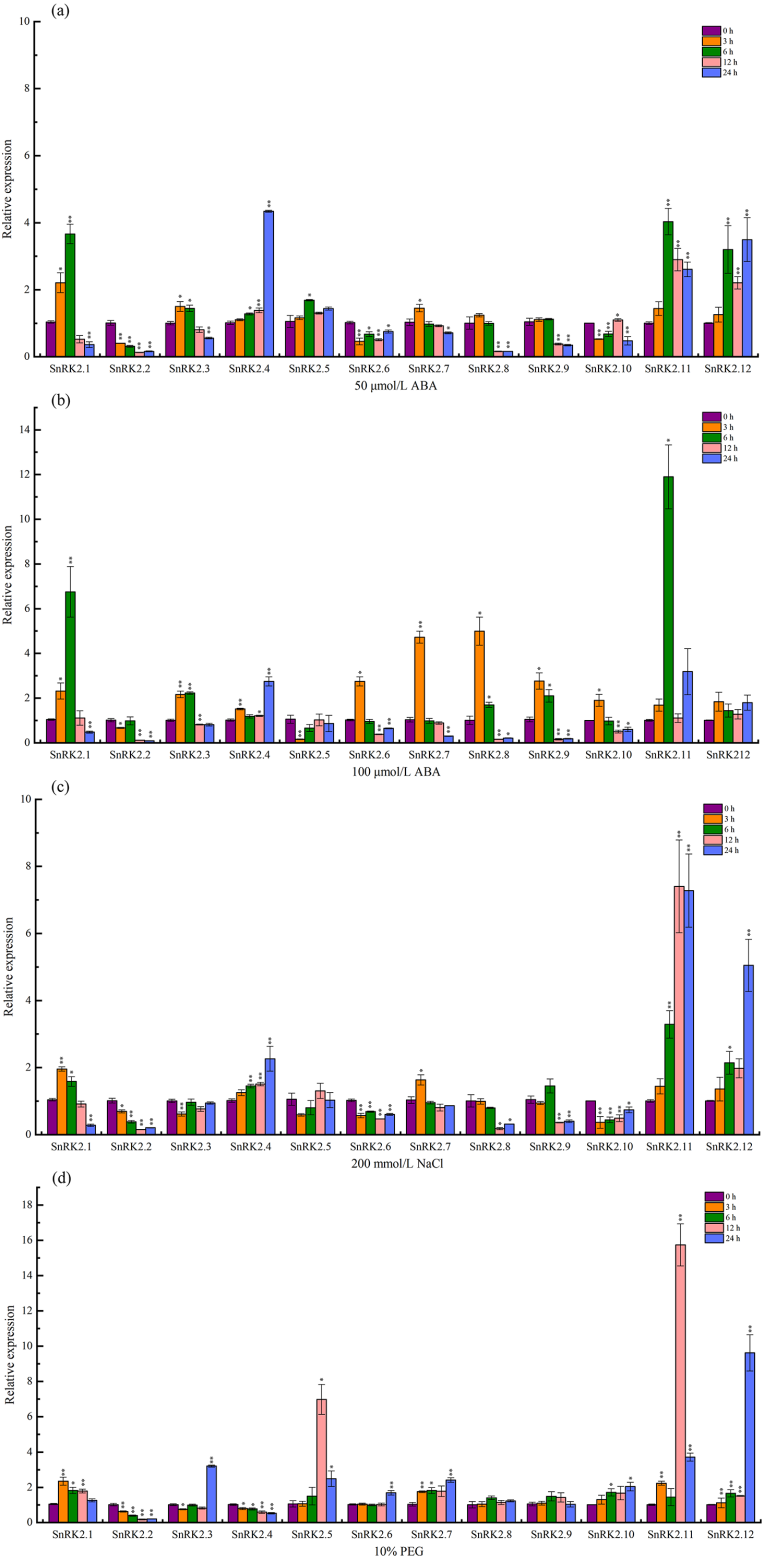
Real-time fluorescence quantitative expression analysis of *CsSnRK2* genes. (A) A total of 50 mmol/L ABA, (B) 100 mmol/L ABA, (C) 200 mmol/L NaCl, and (d) 10% PEG. The legend numbers 3 h, 6 h, 12 h and 24 h indicate the sampling time, and 0 h is set as the control. With CsActin as the internal parameter, the relative expression was calculated by 2^- ΔΔCt^ method, and value represents mean ±SE of the three biological replications. Asterisks indicated values that are significantly different from CK (0 h) (* *p* < 0.05, ** *p* < 0.01, one-way ANOVA).

The expression levels of the *CsSnRK2* genes under salt, PEG, and ABA stress were determined using qRT-PCR. A total of 50 µmol/L ABA (T1), 100 µmol/L ABA (T2), 200 mmol/L NaCl (T3), and 10% PEG (T4) were sampled at 3, 6, 12, and 24 h, respectively, and 0 h was used as a control. The expression of *CsSnRK2* was significantly different between the different time points and treatments (Fig. 7).

Under 50 µmol/L ABA treatment (Fig. 7A), four genes (*CsSnRK2.4/5/11/12*) were up-regulated, of which *CsSnRK2.11,* and *CsSnRK2.12* were up-regulated the most, and their relative expression levels at 6 and 24 h were 4 and 3.5 times higher than that at 0 h, respectively. *CsSnRK2.4* was highly expressed at 24 h, and its relative expression level at 24 h was 4.3 times higher than that at 0 h. In addition, five genes (*CsSnRK2.2/6/8/9/10*) were down-regulated, and *CsSnRK2.1/3/7* were up-regulated first and then down-regulated. *CsSnRK2.3/7* genes reached their maximum expression values at 3 h.

Upon 100 µmol/L ABA treatment (Fig. 7B), all the genes were up-regulated and then down-regulated, except for *CsSnRK2.2* and *CsSnRK2.5*, which were downregulated. All three genes (*CsSnRK2.4/11/12*) were up-regulated, of which *CsSnRK2.11* was up-regulated the most, and the relative expression level at 24 h was 11.9 times higher than that at 0 h. The expression of *CsSnRK2.1* was the highest at 6 h, and the relative expression levels at 6 h was 6.8 times higher than that at 0 h. The maximum expression of *CsSnRK2.2/6/7/9/10* was reached after 3 h; however, the expression of *CsSnRK2.2* was down-regulated by both ABA treatments, suggesting that *CsSnRK2.2* may play a negative regulatory role.

In response to 200 mmol/L NaCl treatment (Fig. 7C), three genes (*CsSnRK2.4/11/12*) were up-regulated, of which *CsSnRK2.11* and *CsSnRK2.12* were up-regulated the most; their relative expression levels at 12 and 24 h were 7.3 and 5 times higher than that at 0 h, respectively. In addition, five genes (*CsSnRK2.2/6/8/9/10*) were down regulated. The genes *CsSnRK2.1* and *CsSnRK2.7* were up-regulated first and then down-regulated.

For the 10% PEG treatment (Fig. 7D), six genes (*CsSnRK2.1/5/7/10/11/12*) were up-regulated, where *CsSnRK2.11* and *CsSnRK2.12* were up-regulated the most; their relative expression level at 12 and 24 h were 15.7 and 9.6 times higher, respectively, that of 0 h. However, two genes (*CsSnRK2.2/4*) were also down-regulated. The expression of *CsSnRK2.5* reached a maximum at 12 h, and its relative expression level at 12 h was 6.6 times higher than that at 0 h.

This study provides an overview of *CsSnRK2* expression under ABA, NaCl, and PEG stress conditions, where different *CsSnRK2* members had different expression patterns under these stressors. This illustrates that the functional diversity of this gene is widely present in different plant species.

### Analysis of the protein interaction network of the *CsSnRK2* family genes

A protein–protein interaction network between CsSnRK2 and other cucumber proteins was constructed using the STRING database. According to the prediction results, cucumber SnRK2 protein can interact with five other proteins (XP_004146814.1, XP_004148120.1, XP_004167207.1, XP_004154794.1, and XP_004168589.1), the first four of which are PP2C proteins, which belong to the PP2C superfamily, and the fifth is a sucrose non-fermentable protein, presumably belonging to the other subfamilies outside SnRK2. The results also showed that eight CsSnRK2 proteins interacted with XP_004146814.1 (PP2C2), XP_004148120.1, and XP_004167207.1. The XP_004154794.1 protein interacted with five CsSnRK2 proteins. In addition, no interaction between CsSnRK2 proteins were found in the prediction results; however, the CsSnRK2 protein interacted with a sucrose non-fermenting protein (XP_004168589.1). We speculated that this protein belongs to a protein family other than SnRK2 and plays an important role. These results provide useful information for further analysis to verify the function of *CsSnRK2* genes.

## Discussion

Plants evolve and develop with changes in the environment, gradually forming a self-protection mechanism against abiotic stress. Under adverse conditions, this protective mechanism can activate the expression of relevant genes and change the structure of relevant functional proteins to protect the normal metabolic response in cells (Bohnert et al., 2006). The plant hormone pathway plays a key role in the ability of plants to adapt to stressful environment (Fujita et al., 2006; Verma, Ravindran & Kumar, 2016). ABA is an important plant hormone with many physiological functions in modulating plant growth and development, salt stress, water deficit, and other stress responses (Golldack et al., 2013; Zhang et al., 2006). In these processes, stress-induced phosphorylation of protein kinases plays a key role in plant perception and response to the environment (Fujita et al., 2006; Wei et al., 2017). SnRK2 is a plant-specific serine/threonine kinase that is activated in response to biotic and abiotic stresses in plants (Coello, Hey & Halford, 2011).

The SnRK2 gene family has been identified in many species, but only a few genes have been functionally validated. Comprehensive and systematic studies on the Cucurbitaceae are limited; thus, the analysis of *CsSnRK2* gene function and characterization deserves specific attention. In this study, 12 *CsSnRK2* genes were identified from the cucumber genome database and named *CsSnRK2.1*-*CsSnRK2.12* (Table 1). Chromosome distribution and protein physical and chemical property analysis revealed that 12 *SnRK2* genes were unevenly distributed on five cucumber chromosomes (chromosomes 2/3/4/6/7), and most of genes were distributed on chromosome 4 (Fig. 1). The three main causes of gene family expansion are tandem replication, fragment replication, and genome-wide replication, which may also produce variations in genetic material that are better adapted to the pressures of natural selection during evolution (Cronk, 2001; Wei, Wang & Xie, 2014). BLAST using MCScanx provided by TBtools revealed no collinear relationship between *CsSnRK2* genes; however, the presence of tandem duplication in multiple genes on chromosome 4 remains to be verified. In addition, the collinearity between *A.thaliana*, maize, rice, and cucumber was analyzed (Fig. 2). There were three pairs of collinear genes between cucumber and *A.thaliana*, four pairs with rice, and one pair with maize. We hypothesized that these genes may be derived from a common ancestor and contribute to the cucumber SnRK2 gene family. Further analysis of the role of evolutionary constraints showed that the d_N_/d_S_ values of all three gene pairs were <1 (Table 4), indicating that the evolutionary process underwent purification selection pressure and was highly conservative, which helped maintain the basic functions of the genes.

Phylogenetic analysis and comparisons were used to understand the evolutionary relationships between genes and species. Cucumber *SnRK2* genes could be divided into three groups (Fig. 3), which is consistent with studies on other species, except for soybean (Wei et al., 2017). CsSnRK2 had high homology with the protein of dicotyledon SnRK2 and was far away from monocotyledons; collinearity analysis showed that *CsSnRK2* had relatively few collinear gene pairs with monocotyledon maize and rice and dicotyledon *Arabidopsis*. These results suggest that *SnRK2* may have evolved from a common ancestor and rapidly diverged after the segregation of monocotyledons and dicotyledons. This further indicated that *CsSnRK2* genes have a relatively conserved evolutionary process.

The structure, length, and number of introns and exons are important features and traces of the evolution of certain gene families (Chen et al., 2013). According to our analysis, the number and size of *SnRK2* exons were highly conserved between cucumber and *Arabidopsis*, with some exceptions. Structural analysis of *CsSnRK2* genes showed two patterns, most of which had nine CDS and eight introns (Fig. 4B). The former gene structure pattern was consistent with that of cotton, and rice (Liu et al., 2017; Saha et al., 2014); however the exons and introns of *CsSnRK2* genes in the third group were different from those in the first and second groups, which may be related to the functional diversity of *SnRK2* genes in cucumber. Previous studies have shown that genes with fewer introns are more highly expressed (Chung et al., 2006). In the present study, *CsSnRK2* gene expression levels were higher in those with fewer introns, which is consistent with previous studies. In addition, the length of *CsSnRK2* gene introns was generally higher than that of *Arabidopsis*; therefore, we speculated that the introns of the *CsSnRK2* genes may be the imprinting that led to the evolutionary process and functional diversity of this gene family.

The SnRK2 family has a highly conserved N-terminal structural domain and C-terminal regulatory structural domain, and the functional diversity of the SnRK2 gene is closely related to its C-terminus (Kulik et al., 2011). The C-terminal domain activates related kinases through protein interactions in response to ABA and signaling (Vlad et al., 2009). In our study (Fig. 4C), we found that groups I and II contained motifs 1–10, except for *AtSnRK2.7* which did not contain motif 9. In addition, motif 9 was missing in the third group *CsSnRK2.7* and *CsSnRK2.8*, while *CsSnRK2.9* was missing motif 4/5/6. Previous reports have demonstrated that there are differences in gene structure and physical and chemical properties among gene families, which may be due to the structural and property changes of gene families to better adapt to the environment during the evolutionary process (Jaramillo & Kramer, 2007; Thornton Joseph & De Salle 2000). Therefore, we hypothesized that motifs 1/2/3/7/10 are important components of the C-terminus of the amino acid sequence of the *CsSnRK2* gene family. A few gene motif deletions may be due to structural polymorphisms in SnRK2 during long-term evolution, different evolutionary pathways, and some functional divergence.

When plants are stressed, through a series of signal transductions, the plants themselves produce resistance factors to activate the related transcription factors in the plant body and corresponding cis-regulatory elements and activate gene expression to respond to stress (Ali & Komatsu, 2006; Hadiarto & Tran, 2011). A variety of cis-regulatory elements were found in the promoter region of the *CsSnRK2* gene in this study (Fig. 5). A previous study has shown that the combination of multiple ABRE, or one ABRE and the corresponding DRE/CRT elements, can lead to the expression of ABA genes (Fujita, Yoshida & Yamaguchi-Shinozaki, 2013). Our results showed that all genes, except *CsSnRK2.7* and *CsSnRK2.8*, contained ABRE (Fig. 4). However, previous studies have shown that not all genes induced by specific stressors have corresponding cis-acting elements in their promoters (Zhang et al., 2016). For instance, the *CsSnRK2.7* and *CsSnRK2.8* promoters, which do not contain ABRE, could also be induced by ABA (Fig. 6). These results suggest that there may be other unknown stress-related cis-acting elements or mechanisms involved in the regulation of these genes.

Studies have shown that in *Arabidopsis*, the first group of *SnRK2B* is expressed in a variety of tissues (Fujii, Verslues & Zhu, 2007). *GmSnRK2.14* and *GmSnRK2.15* were highly expressed in late soybean seed development, and *GmSnRK2.2* and *GmSnRK2.16* were preferentially expressed in meristematic tissues (Wei et al., 2017). We analyzed the expression levels of the *CsSnRK2* gene family in different tissues using online transcriptome RNA-Seq data (Fig. 6). *CsSnRK2* showed a specific expression pattern in each group. *CsSnRK2.5* and *CsSnRK2.7* were highly expressed in the roots and *CsSnRK2.11* was highly expressed in tendrils and tendril bases. The expression of *CsSnRK2.1*, *CsSnRK2.6*, and *CsSnRK2.7* in the first group was almost undetectable in all tissues. *CsSnRK2.2/4/8/10/12* were highly expressed in all tissues, and their function may have an important relationship with growth and development. In our gene expression analysis, *CsSnRK2* expression in the leaf tissues differed from that in the transcriptome RNA-Seq data. For example, *CsSnRK2.1* was induced by ABA, PEG, and NaCl to upregulate its expression. *CsSnRK2.8* has only three elements, LTR, P-box, and MBS, which are involved in low temperature, gibberellin, and drought-inducible elements, respectively; however, *CsSnRK2.8* was moderate to highly expressed in various tissues (Fig. 6). These results suggest that the activity of the *CsSnRK2* genes is not specifically associated with region differences.

Previous studies have shown that *SnRK2* genes in *Arabidopsis* can be clustered into three groups. Groups I and II are activated under hyperosmotic stress, and members of groups II and III responded to both ABA and hyperosmotic signals (Nakashima et al., 2009). In particular, the SnRK2 gene family in the third group was highly induced by ABA and is an important component in the response to ABA signaling (Yamaguchi-Shinozaki, 2010; Yoshida et al., 2006). In the present study, *CsSnRK2.1* and *CsSnRK2.12* in the third group were strongly induced by ABA (Figs. 7A, 7B). In the second group, *CsSnRK2.11* was strongly induced by ABA. The third group of *CsSnRK2* showed a weak ABA response. These results are not entirely consistent with those of previous studies in *Arabidopsis*, where the group III members *AtSnRK2.2*, *AtSnRK2.3*, and *AtSnRK2.6*, were strongly induced by ABA (Kobayashi et al., 2004). Some *SnRK2* genes are the main regulatory factors in ABA signal transduction, but some may not be affected by ABA and have acquired unique regulatory properties (Boudsocq, Barbier-Brygoo & Lauriere, 2004; Kobayashi et al., 2004). Thus, the fact that the third group of *CsSnRK2.2* was not activated by ABA, indicates a specific role of *CsSnRK2.2* in response to ABA signaling. All *TaSnRK2* members are rapidly induced by PEG and NaCl treatments (Zhang et al., 2016). In this study, *CsSnRK2.11* and *CsSnRK2.12* of the second and third groups were strongly induced by PEG and NaCl (Figs. 7C, 7D). Moreover, the elements involved in the drought-induced response (MBS) and the cis-acting elements (TC-rich repeats) involved in defense and stress response were also found in the promoter, indicating that these elements may be responsible for the co-expression of *CsSnRK2* in the stress response of cucumber under different environmental conditions (Fig. 4). The first group of *CsSnRK2* was weakly responsive to PEG and NaCl treatments, except for *CsSnRK2.4* and *CsSnRK2.5* which were strongly activated by NaCl and PEG, respectively. The third group of *CsSnRK2.2* was not activated by drought and salt stresses, suggesting that *CsSnRK2.2* plays a special role in responding to hyperosmotic signals. Therefore, these results are not entirely consistent with the other *Arabidopsis* studies (Saha et al., 2014). Taken together, the expression profile of *CsSnRK2* under abiotic stress and ABA treatment suggests that *SnRK2* genes have conserved and diverse biological functions in the plant kingdom.

**Figure 8:**
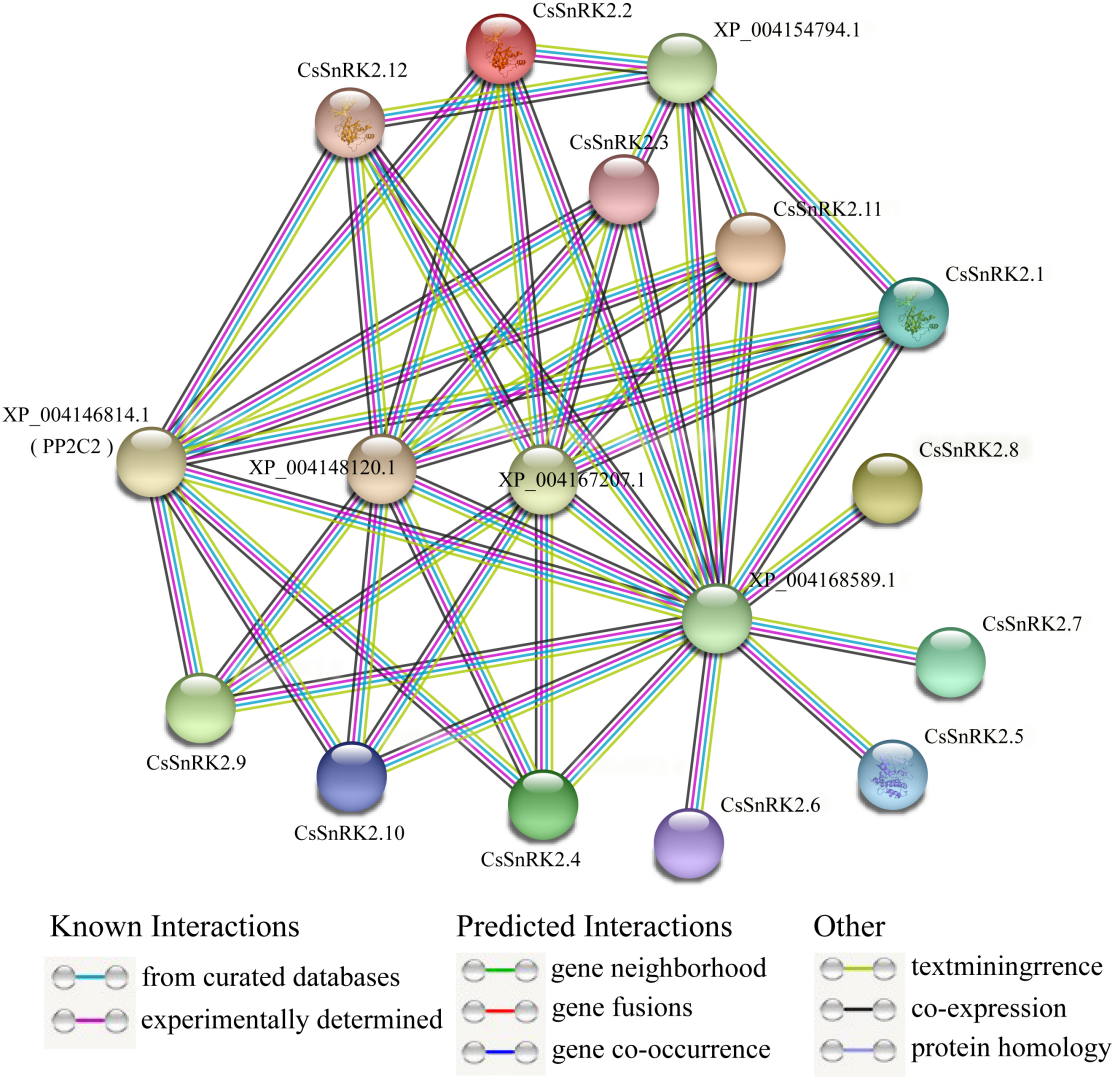
Interaction network analysis of CsSnRK2 protein. Protein–protein interaction networks were predicted using STRING. Each node represents a protein, and each edge represents an interaction, colored by evidence type.

PP2C and SnRK2 are second and third messengers of the ABA signal transduction pathway. The inactivation of PP2C phosphatase allows SnRK2 inhibition to be lifted, activating the phosphorylation mechanism and catalyzing downstream transcription factors that regulate plant resistance to adversity (Ma et al., 2009; Soon et al., 2012). Studies on rice *OsPYL3* have revealed that *OsPYL3* can interact with some class A *OsPP2C* genes and that transgenic rice seeds have enhanced tolerance to cold and drought stresses during germination (Tian et al., 2015). In *Arabidopsis*, type A PP2C of regulates ABF, SLAC1, and other components in the downstream signaling pathway, by inhibiting the activity of *AtSnRK2.2*, *AtSnRK2.3* and *AtSnRK2.6* protein kinases; thus mediating the response to stress (Fujita et al., 2009). In one study, three *CsPYL*, four *CsPP2C,* and two *CsSnRK2* genes were obtained by homologous cloning. Phylogenetic tree analysis of *Arabidopsis* and cucumber genes showed that *CsPP2C2* belonged to group A PP2C and *CsSnRK2.2* belonged to group III, and these two genes were hypothesized to be involved in ABA signal transduction (Wang et al., 2012). In our prediction (Fig. 8), multiple *CsSnRK2* proteins interacted with four proteins, including XP_004146814.1 (PP2C2) and another PP2C, and all of them responded to ABA treatment to varying degrees (Fig. 7); for example, *CsSnRK2.11/12* responded strongly to ABA. This suggests that the *CsSnRK2* genes may also play an important role as a third messenger in the ABA signaling pathway. In addition, sucrose non-fermentable proteins (XP_004168589.1) which we speculate may belong to a subfamily, other than SnRK2, interact with the *CsSnRK2* protein, and play important roles in some specific regulatory pathways.

## Conclusions

In this study, in a genome-wide search of cucumber, we identified 12 SnRK2 gene family members and analyzed them in detail. These *SnRK2* genes were distributed on five chromosomes, and phylogenetic clustering divided them into three well-supported clades. In addition, collinearity analysis showed that the CsSnRK2 gene family has underwent purifying selection pressure during evolution. *CsSnRK2* genes of the same group had similar exons and conserved motifs, and their intron length may be a specific imprint for the evolutionary amplification of the CsSnRK2 gene family. By predicting cis elements in the promoter, we found that the promoter region of *CsSnRK2* gene members had various cis-regulatory elements in response to hormones and stress. Relative expression analysis showed that *CsSnRK2.11* (group II) and *CsSnRK2.12* (group III) was strongly induced by ABA, NaCl and PEG stress, respectively; whereas *CsSnRK2.2* (group III) was not activated by any treatment. The response of group I *CsSnRK2* to ABA, NaCl and PEG was weak. Protein interaction prediction showed that multiple CsSnRK2 proteins interacted with four proteins, including PP2C, and the *CsSnRK2* genes may also play an independent role as third messengers in the ABA signaling pathway. These results provide a reference for future analysis of the potential function of *CsSnRK2* genes.

##  Supplemental Information

10.7717/peerj.13994/supp-1 Supplemental Information 1 Protein sequences

10.7717/peerj.13994/supp-2 Supplemental Information 2 The original CT value of qRT-PCR

## Additional Information and Declarations

### Data Availability

The following information was supplied regarding data availability: The raw data is available in the Supplementary File.
